# The effects of soil liming and sewage sludge application on dynamics of copper fractions and total copper concentration

**DOI:** 10.1007/s10661-016-5609-4

**Published:** 2016-09-29

**Authors:** Elżbieta Malinowska

**Affiliations:** Department of Grassland and Landscape Architecture, Siedlce University of Natural Sciences and Humanities, B. Prusa 14 Street, 08-110 Siedlce, Poland

**Keywords:** Copper mobility, Sewage sludge, Liming, Soil, Zeien and Brümmer method

## Abstract

The paper deals with effects of liming and different doses of municipal sewage sludge (5, 10, and 15 % of soil mass) on copper speciation in soil. In all samples, pH was determined together with total copper concentration, which was measured with the ICP-AES method. Concentration of copper chemical fractions was determined using the seven-step procedure of Zeien and Brümmer. In the soil treated with the highest dose of sludge (15 %), there was, compared to the control, a twofold increase in the concentration of copper and a threefold increase in the concentration of nitrogen. Copper speciation analysis showed that in the municipal sewage sludge the easily soluble and exchangeable fractions (F1 and F2) constituted only a small share of copper with the highest amount of this metal in the organic (F4) and residual (F7) fractions. In the soil, at the beginning of the experiment, the highest share was in the organic fraction (F4), the residual fraction (F7) but also in the fraction where copper is bound to amorphous iron oxides (F5). After 420 days, at the end of the experiment, the highest amount of copper was mainly in the organic fraction (F4) and in the fraction with amorphous iron oxides (F5). Due to mineralization of organic matter in the sewage sludge, copper was released into the soil with the share of the residual fraction (F7) decreasing. In this fraction, there was much more copper in limed soil than in non-limed soil.

## Introduction

Because of the growing number of sewage treatment plants and the problem of sewage sludge disposal, the most economical way to utilise the growing amounts is its agricultural application (Gambuś and Gorlach 1998; Malinowska et al. 2015). The only factor limiting sewage sludge use in agriculture is the fact that it contains heavy metals and pathogens (Behel et al. 1983; Kalembasa and Malinowska 2013). Thus, the application of organic waste materials to improve soil fertility poses a risk to the environment. Uptake of heavy metals by plants causes their accumulation in the food chain, threatening human and animal health. The following metals are particularly dangerous: lead, zinc, cadmium, and copper. Copper, one of the heavy metals, can cause harmful health effects. Because of that, monitoring of environmental hazards, including, first of all, monitoring of soil, a major element of food chain, is of crucial importance. Speciation analysis makes it possible to determine potential hazards of heavy metals present in sewage sludge and in soil to a given ecosystem (Sauvé et al. 1997; Glyzes et al. 2002; Rao et al. 2008; Yang et al. 2009). Availability of different soil metals to plants can be determined by using the sequential extraction procedure together with physical speciation (McBride et al. 1997; Kalembkiewicz and Sočo 2005; Kelderman and Osman 2007; Yang et al. 2009; Vollmannová et al. 2011).

Sequential extraction is based on using different chemical solutions, called extraction reagents, of increasing ability to extract a whole spectrum of fractions, from metals loosely bound to soil components (soluble and labile forms) to fractions strongly bound to soil and, this way, not immediately harmful to the environment (Singh et al. 1995).

For the needs of environmental protection and food production, it is necessary to determine toxicity of heavy metals present in soil, no matter what their origin is. Such information could be used to set safe limits of the amounts of those substances (Kabata-Pendias and Pendias 1999; Dąbrowska 2011).

The aim of the paper is to evaluate effects of liming and different amounts of sewage sludge on copper speciation in soil.

## Materials and methods

The incubation experiment, lasting 420 days and reduplicated three times, was carried out in a laboratory room. Pots were filled with 3 kg of soil, with the pH_KCl_ of 4.30. The soil was taken from the humus layer with the granulometric composition of silty light loamy sand (according to Polish Society of Soil Science classification). Before the experiment started, the concentration of those metals was determined and it stood as follows (mg kg^−1^): lead—6.03; cadmium—0.12; chromium—2.09; copper—2.21; zinc—19.95; and nickel—1.56. Those concentrations were below the limits set by the Polish Ministry of the Environment Regulation of 2002 for loose soil treated with sewage sludge. Organic carbon concentration in the soil was 31.3 g kg^−1^, while total nitrogen concentration was 1.45 g kg^−1^. The pots were filled after the soil was sieved through a mesh and divided into two parts, one was not limed but the other was limed with CaCO_3_ of hydrolytic acidity of Hh = 1 and was left for 1 month with the moisture ranging from 50 to 60 % of maximum water holding capacity. Every 3 days deionised water was used to replace evaporated water and to keep the soil moisture content stable. This way there were two groups of pots: with non-limed soil and with limed soil. Next, fresh sewage sludge from the mechanical and biological sewage treatment plant in Siedlce was added in different doses of 150, 300, and 450 g, which constituted 5, 10, and 15 % of the dry mass of the soil, respectively. In that treatment plant, the activated sludge process with increased nutrient removal was used to treat wastewater. The sludge was dewatered with the gravity thickener, then it underwent methane fermentation and was, in part, dehydrated mechanically. Next, the contents were thoroughly mixed. Heavy metal concentration in the sludge did not exceed the limits set by the Polish Ministry of the Environment Regulation of 2010 (Table 1). The dry matter of the sewage sludge was determined with the oven drying method with the samples dried at 105 °C until a stable weight was reached.

**Table 1:** Concentration of chemical elements and pH of the sludge

DM %	pH	C_org_ g kg^−1^	C:N	N	P	K	Ca	Mg	S	Na
				g kg^−1^ dm						
25.0	6.8	345	8.6:1	40.00	21.23	2.78	38.56	8.41	5.23	0.854
Fe	Mn	B	Mo	Co	Pb	Cd	Cr	Cu	Zn	Ni
mg kg^−1^ dm										
7984	521	8.96	4.21	3.80	50.23	0.174	19.85	85.01	1120	50.14

Two controls were used in the experiment: one without liming and without sludge and the other with liming (CaCO_3_) and also without sludge. During the experiment, moisture was kept at 50–60 % of maximal water capacity and the temperature was 20–22 °C.

Soil samples were taken six times, every 30 days at the beginning and later every 60 days, i.e., after 30, 60, 90, 120, 360, and 420 days. After 120 days had passed, samples were not taken at all for 180 days but the air temperature and soil moisture stayed the same. In all the samples, total copper concentration was measured after dry mineralization at the temperature of 450 °C with the ICP-AES method, while pH was determined with the potentiometric method in 1 mol dm^−3^ KCl. Total nitrogen was determine with the Kjeldahl method, while copper fractions with the seven-step Zeien and Brümmer (1989) method (Table 2).

**Table 2:** Sequential extraction of heavy metals with the Zeien and Brümmer method

Fraction	Name	Extraction reagent	Extraction time	pH
F1	easily soluble	1 mol NH_4_NO_3_ dm^−3^	24 h	natural
F2	exchangeable	1 mol CH_3_COONH_4_ dm^−3^	24 h	6.0
F3	bound to MnO_x_	1 mol NH_2_OH HCl dm^−3^ + 1 mol CH_3_COONH_4_ dm^−3^	0.5 h	6.0
F4	F_org_ bound to organic matter	0.025 mol C_10_H_22_N_4_O_8_. dm^−3^	1.5 h	4.6
F5	bound to amorphous FeO_x_	0.2 mol (NH_4_)_2_C_2_O_4_ dm^−3^ + 0.2 mol H_2_C_2_O_4_ dm^−3^	4 h	3.25
F6	bound to crystalline FeO_x_	0.2 mol (NH_4_)_2_C_2_O_4_ dm^−3^ + 0.2 mol H_2_C_2_O_4_ dm^−3^ + 0.1 mol C_6_H_8_O_6_ dm^−3^	0.5 h	3.25
F7	F_resid_ residual	Calculated as the difference between the total content of a particular heavy metal and the sum of the above determined fractions	–	–

Soil proportion/solution 1 g:10 cm^3^

Achieved results were statistically processed; differences between mean values were estimated applying variance analysis (The Statistica programme, Version 10.0 StatSoft, was applied). Tukey’s test was used to compare means. The straight correlation coefficients between nitrogen, pH, and total copper content and copper fractions in the soil were calculated.

## Results and discussion

In the incubation experiment, the addition of sewage sludge to the soil caused a significant increase of the total nitrogen content. The increase was similar both in limed and non-limed soil (Table 3). The nitrogen content in the soil increased together with higher doses of sewage sludge. Thus, under the influence of the largest doses of sewage sludge, there was a threefold increase in the nitrogen content in the soil in comparison to the control. Many authors hold that there is a significant impact of sludge on the rebuilding of total nitrogen and organic matter (Speir et al. 2003; Malinowska and Kalembasa 2012).

**Table 3:** Total concentration of nitrogen (mg kg^−1^) in soil in the incubation experiment

Days	0	5 %	10 %	15 %	Mean	0	5 %	10 %	15 %	Mean
	without liming					liming				
30	0.958	1.75	2.05	2.89	1.91	0.950	1.85	2.48	3.01	2.07
60	0.967	2.08	2.18	3.04	2.07	0.981	2.05	2.70	3.18	2.23
90	1.02	1.96	2.25	3.15	2.09	1.00	2.14	2.84	3.20	2.30
120	0.985	1.90	2.38	2.90	2.03	0.963	2.09	2.65	3.11	2.20
360	0.966	1.90	2.09	3.15	2.03	0.940	2.13	2.90	3.05	2.26
420	0.945	2.25	2.20	3.26	2.16	0.971	2.41	2.78	3.17	2.33
mean	0.974	1.97	2.19	3.07	2.05	0.967	2.11	2.73	3.12	2.23
LSD_0. 05_ for:	A = 0.116	B = 0.062	C = 0.159							
	A/B = 0.165	B/A = 0.125	A/C = n.s.	C/A = n.s.	B/C = n.s.	C/B = n.s.				

n.s.- not significant difference; 0-control object, 5%, 10%, 15%  of sewage sludge to dry mass of soil; A-sewage sludge dose; B-liming; C-days; A/B, B/A, A/C, C/A, B/C, C/B interaction.

The experiment showed a significant differentiation of total copper content in the soil under the influence of the addition of sewage sludge and liming (Table 4). Throughout the experiment in the soil with the highest doses of sewage sludge (15 %), the content of this metal was more than twofold higher than in the control. Copper concentration ranged between 1.39 and 6.90 mg kg^−1^, with the average not exceeding 6 mg kg^−1^ of the soil DM.

**Table 4:** Total concentration of copper (mg kg^−1^) in soil in the incubation experiment

Days	0	5 %	10 %	15 %	Mean	0	5 %	10 %	15 %	Mean
	without liming					liming				
30	1.50	3.28	3.99	5.29	3.52	1.39	3.19	3.59	4.69	3.22
60	2.09	3.35	3.94	5.85	3.81	2.01	3.63	4.24	5.96	3.96
90	2.25	3.81	6.71	5.53	3.58	2.21	5.57	4.34	5.23	4.34
120	2.48	3.94	5.28	6.90	4.65	2.52	4.41	5.48	5.80	4.55
360	1.65	4.41	4.79	6.01	4.22	1.59	3.91	4.93	5.16	3.90
420	1.73	3.34	4.26	5.14	3.62	1.66	3.15	4.09	5.74	3.66
mean	1.95	3.69	4.83	5.79	4.07	1.90	3.98	4.47	5.43	3.95
LSD_0.05_ for:	A = 0.183	B = 0.098		C = 0.249						
	A/B = 0.259	B/A = 0.197		A/C = 0.259	C/A = 0.288	B/C = 0.241		C/B = 0.353		

*n.s.* not significant difference; 0—control object, 5, 10, and 15 % of sewage sludge to dry mass of soil; A—sewage sludge dose; B—liming; C—days; A/B, B/A, A/C, C/A, B/C, C/B interaction

According to Kalembasa et al. (2011), the total concentration of copper in upper layers of different soils of East-Central Poland ranges from 1.59 to 4.99 mg kg^−1^. However, the content of this metal in soils of Southern Poland is twofold higher (Terelak et al. 1997). Excessive concentration of copper, reaching several hundred mg kg^−1^, may occur in soil contaminated by copper mining and its metallurgy or in areas where copper alloys are produced (Roszyk and Szerszeń 1988). Throughout the experiment, copper concentration in all variants did not differ from normal levels (Kabata-Pendias and Pendias 1999) not exceeding the permissible amounts in agricultural soil set by the Polish Ministry of the Environment Regulation of 2002 (Polish Regulations 2002).

**Fig. 1:**
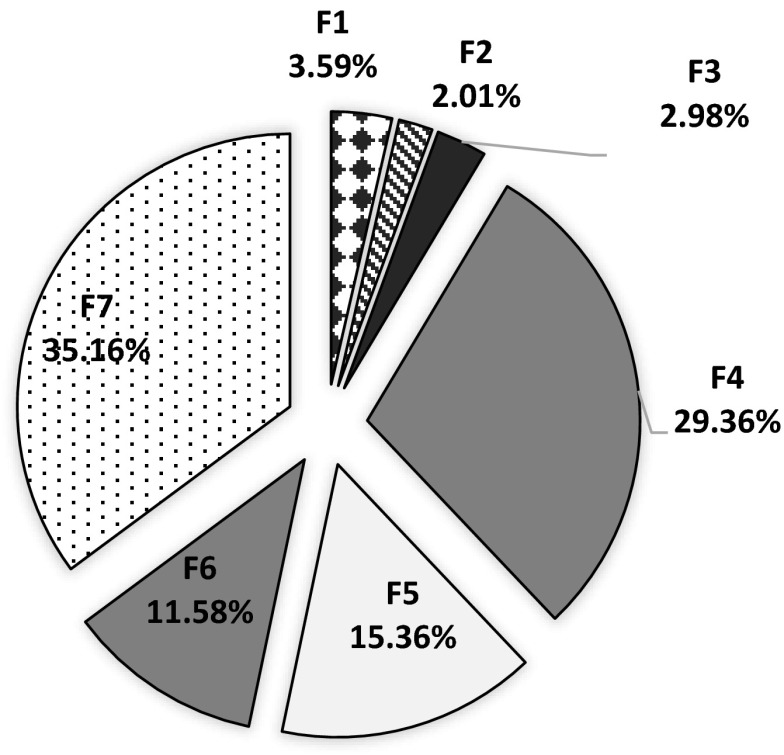
Percentage share of copper fractions in total content in sewage sludge. F1—easily soluble, F2—exchangeable, F3—bound to MnO_x_, F4—bound to organic matter, F5—bound to amorphous FeO_x_, F6—bound to crystalline FeO_x_, F7—residual

Total concentration of copper in the sewage sludge (Table 1) was similar to that provided by other publications (Gondek 2006). Copper speciation in municipal sewage sludge (Fig. 1), determined with the Zeien and Brümmer method, (Zeien and Brümmer 1989) showed that the highest amount of this metal was in the residual fraction F7 (35.16 %) and in the organic fraction F4 (29.36 %). Domination of these fractions in sewage sludge, measured in accordance with the four-step BCR method, was confirmed by Rosik-Dulewska (2003), Wang et al. (2005), Chen et al. (2008), and Gawdzik and Latosińska (2012). Working on sewage sludge Patorczyk-Pytlik and Gediga (2009) also used the BCR method and found a much higher concentration of copper in the residual fraction. In the present experiment, bioavailability of copper in the sludge was low, with F1 and F2 fractions constituting only 4.99 %. Other publications, like Álvarez et al. (2002), Wang et al. (2006), and Latosińska and Gawdzik (2010), confirm that in sewage sludge the content of copper available to plants is low. Taking the findings into account, it can be concluded that non-mobile forms prevailed in the sludge used in the experiment. Copper bound to manganese oxides (F3) constituted 2.98 %, bound to amorphous iron oxides (F5) 15.36 %, while copper bound to crystalline iron oxides (F6) constituted 11.58 % of the total content.

Sequential analysis of copper in the soil showed a wide variation of compounds in which the metal is present, with the content of these compounds depending on the doses of sewage sludge and liming (Tables 5 and 6). Speir et al. (2003) and Hlavay et al. (2004) confirm that both sewage sludge and compost made with sewage sludge cause a change in the mobility of copper in soil. Throughout the present experiment, the highest percentage of mobile forms of copper was noted in the soil where the highest doses of sewage sludge were applied. The amount of mobile forms was higher in the non-limed soil than in limed soil, being also higher for bigger doses of sludge. The sum of the easily soluble (F1) and exchangeable fractions (F2) in the soil with the highest doses of sewage sludge was two to three times higher than in the soil from control pots. According to Gondek (2003), copper in soil fertilised with sludge is more available than in soil fertilised with manure. In the present experiment, the lowest share of the total amount of copper constituted the fraction bound to manganese oxides (F3). The contribution of this fraction to total amount did not exceed 0.35 % and was a little higher in non-limed soil than in limed soil where CaCO_3_ was applied. Compared to the control, sewage sludge application did not cause any significant changes in the content of copper bound to manganese oxides. The largest share of the total amount of copper in the soil represented copper bound to organic matter (F4), mainly at the beginning of the experiment. Copper in this fraction constituted, on average, over 30 % of the total amount and decreased throughout the experiment. In the limed soil sampled after the first 30 days of the experiment, the share of copper bound to organic matter was 43.01 % of the total amount, while in the soil sampled after 120 days it was 30.66 %, while in the non-limed soil it stood at 39.08 and 25.84 %, respectively. After 360 days of the experiment, the share of copper bound to organic matter was over 30 % of the total. The particular affinity of copper for binding to organic matter was also studied and discussed by Ramos et al. (1994) and Kalembasa and Pakuła (2006).

**Table 5:** Percentage share of copper fractions in total content in soil in the incubation experiment

Fertilisation object	F_1_	F_2_	F_3_	F_4_	F_5_	F_6_	F_7_	pH
30 days								
without liming								
Control object	2.00	1.33	0.200	34.66	19.15	16.37	26.32	4.30
5 %	3.35	1.83	0.183	36.59	14.11	20.58	23.35	4.55
10 %	1.70	3.83	0.326	44.61	12.33	15.31	22.01	5.05
15 %	5.52	4.52	0.340	40.45	13.27	13.59	22.29	5.17
mean	3.14	2.88	0.262	39.08	14.72	16.46	23.49	
liming								
Control object	2.88	0.216	0.288	36.76	10.94	17.55	31.46	6.30
5 %	5.08	0.721	0.157	43.59	16.68	11.28	22.49	6.32
10 %	2.28	4.04	0.251	45.40	13.70	12.73	21.67	6.35
15 %	4.50	3.22	0.265	46.27	10.92	13.37	21.49	6.48
mean	3.69	2.05	0.240	43.01	13.06	13.73	24.28	
60 days								
without liming								
Control object	1.48	1.96	0.144	26.51	34.55	9.31	26.10	4.38
5 %	2.70	1.73	0.149	37.40	38.01	6.90	13.13	4.80
10 %	1.40	2.84	0.152	46.45	14.01	12.49	22.66	5.02
15 %	3.21	2.53	0.137	48.38	11.97	10.43	23.35	5.00
mean	2.20	2.27	0.146	39.69	24.64	9.78	21.35	
liming								
Control object	1.69	0.746	0.099	25.62	30.80	12.14	29.00	6.25
5 %	0.178	0.882	0.110	36.36	24.44	12.68	25.36	6.30
10 %	1.84	2.87	0.189	37.26	20.42	11.22	26.21	6.25
15 %	3.31	2.10	0.101	45.69	18.39	8.28	22.13	6.50
mean	1.75	1.65	0.125	36.23	23.51	11.08	25.68	
90 days								
without liming								
Control object	1.47	1.02	0.311	28.88	26.72	13.33	28.31	4.26
5 %	0.656	1.26	0.210	26.81	37.17	8.95	23.95	5.15
10 %	1.15	0.760	0.209	29.36	34.26	7.50	26.83	5.10
15 %	3.38	1.79	0.669	38.52	38.34	9.26	8.04	5.12
mean	1.66	1.21	0.350	30.89	34.12	9.75	22.03	
liming								
Control object	0.181	0.407	0.226	27.00	26.08	13.08	33.10	6.25
5 %	0.377	0.323	0.072	31.77	24.78	8.64	34.11	6.34
10 %	1.23	0.737	0.207	36.87	38.91	10.97	10.15	6.40
15 %	3.17	1.11	0.201	32.89	44.36	9.45	8.81	6.45
mean	1.24	0.644	0.177	32.38	33.53	10.52	21.54	
120 days								
without liming								
Control object	0.201	0.323	0.363	24.82	23.79	11.77	41.13	4.30
5 %	1.65	1.57	0.381	24.44	43.65	11.24	17.06	5.17
10 %	1.70	1.59	0.303	23.11	36.17	11.06	26.14	5.20
15 %	3.04	1.75	0.406	32.32	41.59	9.30	11.59	5.21
mean	1.65	1.31	0.363	25.84	36.30	10.84	23.73	
liming								
Control object	1.09	0.238	0.317	22.22	23.41	11.51	41.27	6.31
5 %	1.34	0.635	0.431	33.56	36.73	8.55	18.82	6.40
10 %	1.31	1.29	0.237	30.38	36.01	6.75	24.45	6.45
15 %	2.74	1.59	0.293	36.38	38.79	8.83	11.38	6.37
mean	1.62	0.938	0.320	30.66	33.74	8.91	23.86	

F_1_—easily soluble, F_2_—exchangeable, F_3_—bound to MnO_x_, F_4_—bound to organic matter, F_5_—bound to amorphous FeO_x_, F_6_—bound to crystalline FeO_x_, F_7_—residual; 5 , 10, and 15 % of sewage sludge to dry mass of soil

**Table 6:** Percentage share of copper fractions in total content in soil in the incubation experiment

Fertilisation objects	F_1_	F_2_	F_3_	F_4_	F_5_	F_6_	F_7_	pH
360 days								
without liming								
Control object	0.424	0.121	0.485	31.52	39.39	17.15	10.90	4.40
5 %	1.22	1.63	0.181	39.23	43.31	10.52	3.91	4.80
10 %	3.74	1.86	0.418	33.82	45.09	11.48	3.59	5.15
15 %	3.93	2.08	0.316	40.27	39.43	11.43	2.55	5.05
mean	2.33	1.42	0.350	36.21	41.81	12.24	5.24	
liming								
Control object	0.252	0.377	0.189	33.08	40.00	17.92	8.18	6.10
5 %	0.512	1.51	0.128	34.27	47.57	11.13	4.88	6.25
10 %	1.26	1.24	0.365	40.57	42.19	10.10	4.29	6.28
15 %	3.04	1.71	0.271	38.37	43.60	9.17	3.84	6.35
mean	1.27	1.21	0.238	36.57	43.34	12.08	5.30	
420 days								
without liming								
Control object	0.173	0.116	0.056	30.20	36.15	23.05	10.27	4.35
5 %	0.479	1.26	0.240	25.16	47.90	21.58	3.40	4.90
10 %	0.892	1.90	0.305	34.07	46.30	12.60	3.95	5.20
15 %	1.77	2.02	0.486	34.46	45.35	13.04	2.88	5.08
mean	0.829	1.32	0.272	30.97	43.93	17.57	5.13	
liming								
Control object	0.181	0.060	0.181	32.05	37.35	17.53	12.65	6.00
5 %	0.127	0.794	0.127	38.09	41.27	11.81	7.71	6.30
10 %	0.611	1.54	0.196	31.05	50.12	11.17	5.31	6.18
15 %	1.27	1.60	0.331	33.97	47.21	11.52	4.09	6.28
mean	0.547	0.999	0.209	33.79	44.01	13.01	7.44	

F_1_—easily soluble, F_2_—exchangeable, F_3_—bound to MnO_x_, F_4_—bound to organic matter, F_5_—bound to amorphous FeO_x_, F_6_—bound to crystalline FeO_x_, F_7_—residual; 5, 10, and 15 % of sewage sludge to dry mass of soil

Mineralization of organic matter in the sewage sludge caused lowering of residual copper (F7) content and the release of copper into the soil. Throughout the experiment, amounts of this copper noted in the control were much higher than in soil fertilised with sludge. At the beginning of the experiment, the share of residual copper in soil with sludge was, on average, over 20 %, while at the end of the experiment, the same share stood at 5 %. There was a bigger share of the residual fraction in limed soil than in non-limed soil. Kalembasa et al. (2011) found that highly stable organic-inorganic and inorganic forms of copper were dominant. There were high amounts of forms of copper bound to iron oxides, in particular to amorphous oxides (F5).

The share of copper bound to amorphous oxides grew with mineralization of organic matter in the sludge. In the soil sampled after 90 days, it was over 30 % of the total amount of copper, while after 360 days of the experiment it was over 40 %. In turn, the share of copper in the fraction bound to crystalline iron oxides (F6) was much lower, and it was stable throughout the experiment. It was found that after sewage sludge application copper concentration in the residual fraction decreased, while it increased in the organic fraction. The findings confirm that organic matter affects the process of heavy metals adsorption (Jain and Ram 1997).

Correlation coefficient was calculated to determine the correlation between total nitrogen content, copper content, and pH of the soil on the one hand and the content of copper fractions on the other (Table 7). The statistical analysis indicated that in both limed and non-limed soil there was a significant positive correlation between total nitrogen content and its content in the easily soluble, exchangeable, and organic fractions. There was a similar correlation in the case of total copper content. There was a significant negative correlation between nitrogen content and the most stable fractions of copper, but also between copper content and the most stable fractions of this metal.

**Table 7:** Simple correlation coefficients between nitrogen, pH and total copper content, and copper fractions in the soil

	N		Cu		pH	
	−Ca	+Ca	−Ca	+Ca	−Ca	+Ca
F1	0.592^*^	0.350	0.521^*^	0.359	0.386	0.567^*^
F2	0.545^*^	0.609^*^	0.409^*^	0.474^*^	0.514^*^	0.375
F3	0.354	0.133	0.287	0.170	0.302	0.145
F4	0.430^*^	0.536^*^	0.301	0.408^*^	0.224	0.385
F5	0.109	0.260	0.125	0.286	0.236	−0.281
F6	−0.358	−0.660^*^	−0525^*^	−0.717^*^	−0.433^*^	−0.676^*^
F7	−0.427	−0.513^*^	−0.255	−0.448^*^	−0.371	0.166

*p* ≤ 0.05, −Ca—without liming, +Ca—liming, ﻿* - significant difference

According to Kalembasa and Malinowska (2013) liming and the kind and content of organic matter affect heavy metal forms in the soil and high values of simple correlation coefficients confirm it.

In the non-limed soil, there was a significant positive correlation between pH and the exchangeable fraction of copper (F2), while in the limed soil such correlation was between pH and the easily soluble fraction (F1). There was also a significant negative correlation between pH and copper bound to crystalline FeO_x_ (F6), no matter what the pH value was.

## Conclusions

In the course of the incubation experiment, sewage sludge doses and liming considerably diversified total content of copper and nitrogen in the soil. Compared to the control, the content of copper in the soil where the highest dose was applied increased more than twofold, while nitrogen content increased threefold.

Using the seven-step sequential procedure to determine copper content in the sludge, it was found that the highest amount of copper was in the residual (F7) and in the organic fraction (F4), which means that bioavailability of this metal was low.

There was a large diversity of copper forms in soil depending on liming and the dose of sewage sludge. Together with the mineralisation of organic matter in the sludge the percentage of copper in the residual fraction (F7) decreased, on average, from 20 to 5 % of the total, while the amount of copper bound to amorphous iron oxides (F5) increased twofold.

As a result of sewage sludge application, there was an increase in the amount of mobile forms of copper. The sum of the easily soluble (F1) and interchangeable (F2) fractions was from twofold to threefold higher in the soil treated with sewage sludge than in the control, but it did not exceed 10 %, making the content of bioavailable copper low. Liming soil with diversified doses of sewage sludge limited copper mobility.
